# The small regulatory RNA molecule MicA is involved in *Salmonella enterica *serovar Typhimurium biofilm formation

**DOI:** 10.1186/1471-2180-10-276

**Published:** 2010-11-02

**Authors:** Gwendoline Kint, David De Coster, Kathleen Marchal, Jos Vanderleyden, Sigrid CJ De Keersmaecker

**Affiliations:** 1 Centre of Microbial and Plant Genetics, K.U.Leuven, Kasteelpark Arenberg 20, 3001 Leuven, Belgium

## Abstract

**Background:**

LuxS is the synthase enzyme of the quorum sensing signal AI-2. In *Salmonella *Typhimurium, it was previously shown that a *luxS *deletion mutant is impaired in biofilm formation. However, this phenotype could not be complemented by extracellular addition of quorum sensing signal molecules.

**Results:**

Analysis of additional *S. *Typhimurium *luxS *mutants indicated that the LuxS enzyme itself is not a prerequisite for a wild type mature biofilm. However, in close proximity of the *luxS *coding sequence, a small RNA molecule, MicA, is encoded on the opposite DNA strand. Interference with the MicA expression level showed that a balanced MicA level is essential for mature *Salmonella *biofilm formation. Several MicA targets known to date have previously been reported to be implicated in biofilm formation in *Salmonella *or in other bacterial species. Additionally, we showed by RT-qPCR analysis that MicA levels are indeed altered in some *luxS *mutants, corresponding to their biofilm formation phenotype.

**Conclusions:**

We show that the *S. *Typhimurium biofilm formation phenotype of a *luxS *mutant in which the complete coding region is deleted, is dependent on the sRNA molecule MicA, encoded in the *luxS *adjacent genomic region, rather than on LuxS itself. Future studies are required to fully elucidate the role of MicA in *Salmonella *biofilm formation.

## Background

*Salmonella enterica *serovar Typhimurium (*S. *Typhimurium) is an important pathogen causing gastroenteritis in humans [1]. *Salmonella *is able to form biofilms on both biotic and abiotic surfaces. Growth in such biofilm structures increases the resistance against antibacterial treatments and enhances their spread and persistence outside the host [2]. Also, contamination of processed foods in industrial plants is often due to biofilm formation on both food and food-contact surfaces [3].

In some bacterial species, it has been reported that biofilm formation is partially regulated by a communication system called quorum sensing, more specifically depending on the quorum sensing synthase enzyme LuxS and the signaling molecule autoinducer-2 (AI-2) produced by LuxS [4-9]. In the case of *Salmonella *Typhimurium, it has been reported that biofilm formation is affected by mutating the *luxS *gene [10-12]. However, De Keersmaecker *et al*. [10] showed that, although genetic complementation could be accomplished, the biofilm forming phenotype could not be rescued by the addition of synthetic DPD, which non-catalytically is converted to AI-2. This suggested that AI-2 is not the actual signal involved in the formation of a *Salmonella *Typhimurium biofilm. Similarly, Karavolos *et al*. [13] reported altered flagellar phase variation in a *S. *Typhimurium *luxS *deletion mutant independent of quorum sensing signals.

In order to further reveal the exact role of the *luxS *region in *S. *Typhimurium biofilm formation, we analyzed additional *S. *Typhimurium *luxS *mutants for their biofilm phenotype. We show that the *S. *Typhimurium biofilm formation phenotype is dependent on the sRNA molecule MicA, encoded in the *luxS *adjacent genomic region, rather than on LuxS itself.

## Results

### Phenotypic analysis of different *luxS *mutants

**Figure 1:**
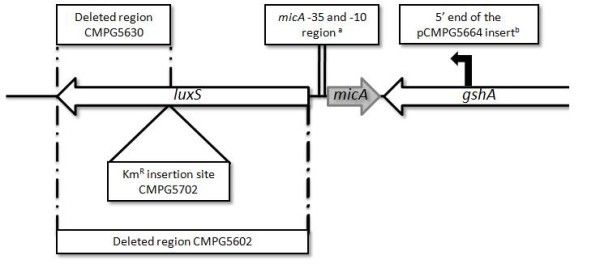
**Genomic organization of the *luxS *region in *Salmonella *Typhimurium**. Coding sequences are depicted with arrows. Mutated regions in different *luxS *mutants are indicated. The figure is drawn to scale. ^a ^The putative -10 and -35 regions of MicA as reported by Udekwu *et al*. [17]. ^b ^5' end of the *luxS *fragment with own promoter for the construction of the complementation construct pCMPG5664 as reported by De Keersmaecker *et al*. [10].

**Figure 2:**
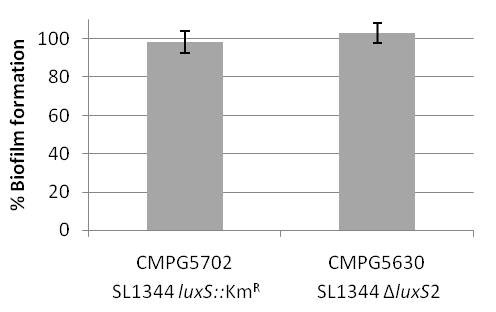
**Biofilm formation of different *Salmonella *Typhimurium *luxS *mutants**. Peg biofilm formation assay of SL1344 *luxS*::Km insertion mutant (CMPG5702) and SL1344 Δ*luxS2 *mutant (CMPG5630). Biofilm formation is expressed as percentage of wildtype SL1344 biofilm. Error bars depict 1% confidence intervals of at least three biological replicates.

Previously, we reported that a *S. *Typhimurium SL1344 *luxS *mutant lacking the entire LuxS coding sequence - from start to stopcodon - (CMPG5602) is unable to form a mature biofilm [10]. This phenotype could be complemented by introduction of the *luxS *gene under control of its own promoter but not by expressing LuxS from a constitutive *nptII *promoter [10]. To further elaborate on this observation, we tested the biofilm formation capacity of other defined *S. *Typhimurium *luxS *mutants. Figure 1 depicts the genomic *luxS *region in *S. *Typhimurium and indicates the genotype differences among the *luxS *mutants discussed in this study. A *S. *Typhimurium *luxS::Km *insertion mutant (CMPG5702, [14]) carrying a kanamycin resistance cassette chromosomally inserted in a *Cla*I restriction site in the *luxS *coding sequence is unable to form AI-2. This is in agreement with the lack of AI-2 production in the deletion mutant CMPG5602 [10,14] and is as expected since both mutants, CMPG5702 and CMPG5602, are unable to form the AI-2 synthase enzyme LuxS, confirmed by western blot analysis with anti-LuxS antibody (data not shown). However, the insertion mutant still makes wildtype biofilm (Figure 2). To eliminate possible polar effects due to the presence of the kanamycin resistance cassette, a second *luxS *deletion mutant was constructed, using the same procedure as for the first deletion mutant CMPG5602. Yet, this second mutant (CMPG5630) only lacks the 3' part of the *luxS *coding sequence starting from the *ClaI *restriction site where the kanamycin cassette was inserted in CMPG5702 (Figure 1). Western blot analysis and AI-2 tests showed that this mutant is unable to form LuxS protein and AI-2 (data not shown). Nevertheless, similarly to the *luxS *insertion mutant, strain CMPG5630 is still able to form a mature wildtype biofilm (Figure 2).

The question then rises which features of the *luxS *genomic region can explain the differences in biofilm formation phenotype between strain CMPG5602 - lacking the entire *luxS *coding sequence - on the one hand and both CMPG5702 and CMPG5630 on the other hand. In *Salmonella *Typhimurium, as in *E. coli*, a small non-coding RNA molecule, termed MicA, is encoded in the opposite strand of *luxS *(Figure 1) [15]. The close proximity of both genes could imply interference with MicA expression when the *luxS *genomic region is mutated. We therefore investigated the possibility that the defect of biofilm formation by CMPG5602 could be due to interference of the *luxS *deletion with MicA expression.

### MicA has an effect on *Salmonella *biofilm formation

**Figure 3:**
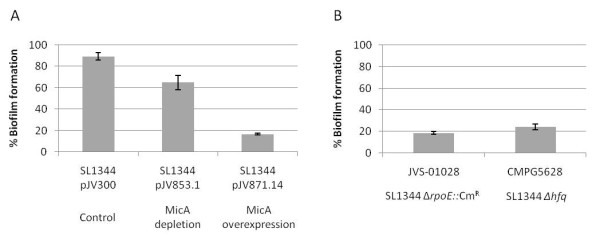
**Biofilm formation of *Salmonella *Typhimurium linked to sRNA**. (A) Biofilm formation assay of *S. *Typhimurium SL1344 containing the control vector (pJV300), MicA depletion (pJV853.1) or overexpression (pJV871.14) constructs. (B) Biofilm formation assay of *S. *Typhimurium SL1344 *rpoE *(JVS-01028) and *hfq *(CMPG5628) deletion mutants. Biofilm formation is expressed as percentage of wildtype SL1344 biofilm. Error bars depict 1% confidence intervals of at least three biological replicates.

To assess the effect of MicA on biofilm formation, two different plasmids were used. The first plasmid, pJV853.1, encodes a MicA antisense sequence, thereby leading to partial depletion of MicA in the cell due to formation of unstable double stranded RNA. The second plasmid, pJV871.14, is a MicA overexpression construct, constitutively expressing MicA from a strong P_LlacO _promoter. The ampicillin resistant pJV300 plasmid used for both constructs, was included as a negative control. All plasmids were electroporated to wildtype *S. *Typhimurium SL1344 and the resulting strains were tested for biofilm formation using the peg system quantifying the formed biofilms with crystal violet [10]. The results are shown in Figure 3A. Interestingly, the presence of either the overexpression or the depletion construct had an impact on the biofilm forming capacity of *S. *Typhimurium although not to the same extent. Biofilm formation was almost completely abolished in the MicA overexpression strain while only slightly, but significantly decreased in the MicA depletion strain. This indicates that a tightly regulated balance of MicA expression is essential for proper biofilm formation in *Salmonella *Typhimurium. Note that all strains with the above plasmid constructs produce wildtype AI-2 levels (data not shown).

Further indirect evidence of small RNA molecules being involved in the regulation of biofilm formation was provided by the analysis of both *hfq *and *rpoE *mutants. Hfq is a prerequisite for the binding of many sRNAs to their *trans*-encoded targets [16,17], while sigmaE, encoded by *rpoE*, has been shown to be involved in the transcription of several small RNAs, including MicA [18-20]. In the peg biofilm assay, neither of these strains were able to form mature biofilms (Figure 3B). The phenotype could genetically be complemented by introducing the corresponding gene *in trans *on a plasmid carrying a constitutive promoter (data not shown).

### MicA targets involved in *Salmonella *biofilm formation

**Figure 4:**
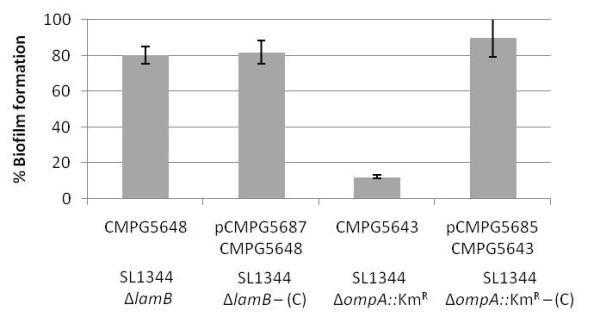
**Biofilm formation of *lamB *and *ompA *deletion mutants in *Salmonella *Typhimurium**. Peg biofilm formation assay of SL1344 Δ*lamB *(CMPG5648) and SL1344 Δ*ompA *(CMPG5643) and the corresponding complementation strains pCMPG5687/CMPG5648 for *lamB *and pCMPG5685/CMPG5643 for *ompA*. Biofilm formation is expressed as percentage of wildtype SL1344 biofilm. Error bars depict 1% confidence intervals of at least three biological replicates. (C) stands for complemented.

Most likely, the impact of MicA on biofilm formation in *Salmonella *is through one of its *Salmonella *targets. To date, four *trans *encoded targets, all negatively regulated by MicA, have already been reported in *Escherichia coli*, i.e. the outer membrane porins OmpA [17,21] and OmpX [22], the maltoporin LamB [23] and recently the PhoPQ two-component system [24]. Two of these targets, PhoPQ and OmpA, were previously shown to be involved in biofilm formation [25-27], i.e. Prouty and Gunn [25] demonstrated that a *S. *Typhimurium *phoP *null mutant has an enhanced biofilm forming capacity, while a PhoP constitutive mutant is unable to develop a mature biofilm. OmpA was shown to be involved in *E. coli *biofilm formation [26,27]. To assess whether OmpA is also implicated in biofilm formation in *Salmonella*, we constructed an *ompA *deletion mutant in *S. *Typhimurium SL1344 and tested this strain with the peg biofilm assay. As in *E. coli*, a *S. *Typhimurium *ompA *mutant is unable to form biofilm, and this phenotype can be complemented by introducing *ompA in trans *(Figure 4). As no information is yet reported on the role of LamB in biofilm formation, we also constructed a *lamB *deletion mutant. The results in Figure 4 indicate that this mutant is not significantly affected in its biofilm forming capacity, confirming that not all MicA targets known to date are implicated in biofilm formation. Note that both the *S. *Typhimurium *lamB *and *ompA *deletion mutant are still capable of forming AI-2 (data not shown).

### Analysis of MicA levels in *S*. Typhimurium *luxS *mutants

**Figure 5:**
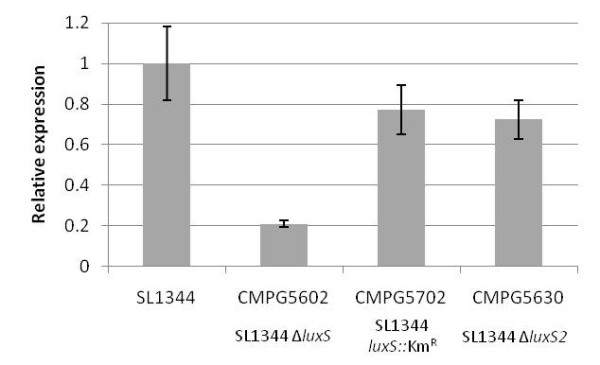
**RT-qPCR analysis of different *S*. Typhimurium *luxS *mutants with MicA primers**. MicA sRNA expression levels were measured with RT-qPCR as described in the Methods section. Representative means and standard deviations of three RT-qPCRs are shown. Gene expression is expressed relative to the wildtype SL1344 level. CMPG5602: SL1344 Δ*luxS *deletion mutant; CMPG5702: SL1344 *luxS*::Km^R ^insertion mutant; CMPG5630: SL1344 Δ*luxS2 *deletion mutant.

From the results described in the previous paragraphs, it can be concluded that the sRNA MicA is indeed implicated in the regulation of biofilm formation in *S. *Typhimurium. The question remains however, whether different MicA levels occur in wildtype and the *luxS *deletion mutant (CMPG5602), thereby explaining the biofilm formation phenotype of the latter. Using RT-qPCR, the amount of MicA was quantitatively assessed in wildtype SL1344, the *luxS *deletion mutant CMPG5602 -unable to form a mature biofilm - and the *luxS *insertion mutant CMPG5702 and partial deletion mutant CMPG5630 - forming a wildtype biofilm, all strains grown under biofilm forming conditions. The entire *luxS *CDS deletion strain CMPG5602 contains significantly less MicA compared to wildtype SL1344. Conversely, both CMPG5702 and CMPG5630, still capable of making biofilm, have a MicA expression level comparable to the wildtype strain (Figure 5). To rule out the possibility that these differential expression levels are due to the difference between biofilm cells (in wildtype) and planktonic cells (in the *luxS *deletion mutant), we performed the experiment also using planktonic wildtype cells from the medium above the biofilm, sampled similarly as for the *luxS *deletion mutant cells (*cf. *Methods section). The relative difference in MicA expression level was similar in this experimental setup, i.e. a significantly lower MicA expression level in the *luxS *deletion as compared to wildtype *S. *Typhimurium (data not shown). Overall, these results confirm that mutating the *luxS *genomic region can have a significant impact on MicA sRNA levels, consequently affecting the MicA regulated biofilm phenotype, independently of quorum sensing.

## Discussion

In several bacteria, biofilm formation capacity has been linked to *luxS *based quorum sensing, mediated by AI-2 signaling molecules [4-9]. In *Salmonella *Typhimurium, it was previously reported that a deletion mutant of the AI-2 synthase enzyme *luxS *has an impaired biofilm formation capacity [10]. However, this phenotype could not be chemically complemented by extracellular addition of synthetic DPD, nor by expressing *luxS *from a constitutive promoter on a plasmid. On the other hand, introduction of *luxS *with its native promoter did complement the biofilm phenotype [10]. In this study, we showed that both a *luxS*::Km insertion mutant and a deletion mutant of the 3' end of the *luxS *coding sequence are still able to form a mature biofilm, despite the fact that these strains are unable to form the type-2 quorum sensing signaling molecule AI-2.

Adjacent to the *luxS *coding sequence, a small non-coding RNA molecule named MicA is encoded in the opposite strand [15]. Using MicA depletion and overexpression constructs, respectively, we showed that a tightly balanced MicA concentration is essential for proper biofilm formation in *S. *Typhimurium. This suggests that the final impact of MicA regulation on biofilm formation is based on a complex interplay of several of its targets, a fine-tuning process in which timing is also likely to play a role. It is interesting to note that the MicA depletion strain does not completely abolish the biofilm formation capacity. This could be explained by an incomplete silencing of MicA in this strain or by the fact that other sRNA molecules take over the role of MicA. It is not uncommon that mRNA targets are redundantly regulated by multiple sRNA molecules fine-tuning their expression in a complex way [28,29]. The fact that deletion of both *rpoE *or *hfq *fully inhibited biofilm formation supports the hypothesis that other sRNA molecules are implicated in regulation of biofilm formation.

In literature, two MicA targets known to date were previously linked to biofilm formation. An *E. coli ompA *mutant is unable to form a mature biofilm on plastic substrates [27]. We showed that also in *Salmonella *Typhimurium, OmpA is involved in biofilm formation as an *ompA *deletion mutant is unable to form a mature biofilm. Furthermore, the two-component system PhoPQ, previously shown to be implicated in regulation of *Salmonella *biofilm formation [25], was recently described as a target of MicA in *E. coli *[24], implying indirect regulation of the entire PhoPQ regulon by MicA. At this moment, it cannot be excluded that other, yet uncharacterized targets of MicA exist which are related to biofilm formation. Nevertheless, it is already clear that MicA regulation comprises a complex network of interactions influencing a broad range of genes either directly or indirectly.

Using RT-qPCR analyses, we were able to confirm that the levels of MicA in the *luxS *CDS deletion mutant CMPG5602 compared to wildtype and the insertion mutant CMPG5702 differ. This supports our formulated hypothesis that an impaired biofilm formation phenotype in a *Salmonella *Typhimurium *luxS *deletion mutant is due to an imbalanced MicA level, rather than to the absence of LuxS itself. Remark that complementation of the CMPG5602 phenotype requiring expression of *luxS *from its native promoter [10] also corroborates with this model (Figure 1). Indeed, MicA is encoded in this promoter region and hence, the biofilm phenotype can only be complemented by reintroduction of MicA.

Presently, it is still unclear how deletion of the *luxS *CDS influences MicA expression. The putative -10 and -35 regions of MicA as reported by Udekwu *et al. *[17] do not overlap with the coding region of *luxS *(Figure 1). However, this coding region might include other regulatory elements interfering with MicA expression. Further studies of both *luxS *and *micA *promoter regions and transcription are required to elucidate the mechanism of interference between both genetic loci.

## Conclusions

In this study, we showed by analyzing different *S. *Typhimurium mutants that biofilm formation is influenced by the sRNA molecule MicA. This sRNA is encoded in close proximity of the quorum sensing synthase *luxS *and mutating this region can therefore mutually affect both genetic loci. Given the evolutionary conservation of MicA in several *Enterobacteriaceae*, this regulatory mechanism of biofilm formation might also apply to bacterial species other than *Salmonella*.

## Methods

### Bacterial strains and growth conditions

The parental strains and plasmids that were used in this study are listed in Table 1. *Salmonella *Typhimurium SL1344 is the wildtype strain [30]. The *Salmonella *Typhimurium *Δhfq *(CMPG5628), *S. *Typhimurium Δ*luxS2 *(CMPG5630) and *ΔlamB *(CMPG5648) mutants were constructed using the procedure of Datsenko and Wanner [31], with pKD3 as a template plasmid (all primers used in this study are listed in Table 2). All strains were verified by PCR and sequencing. For the OmpA and LamB complementation constructs, *ompA *and *lamB *were amplified with PCR using primers PRO-0101/PRO-0102 and PRO-0474/PRO-0475, respectively, and cloned as an *Xba*I*/Pst*I fragment into pFAJ1708 [32]. Both plasmids were verified by PCR and sequencing and finally electroporated to the corresponding SL1344 mutant background.

**Table 1:** Bacterial strains and plasmids

Strains	Description	Source or reference
*Escherichia coli*		
DH5α	F^- ^ϕ80Δ*lacZM15 *Δ(*lacZYAargF)*U169 *deoP recA1 endA1 hsdR17 *(r_k_^- ^m_k_^-^)	Gibco BRL
TOP10F'	F' {*lacIq *Tn10(TetR)} *mcrA *Δ(*mrr-hsdRMS-mcrBC*) ϕ80*lacZΔM15 ΔlacX74 deoR recA1 araD139 *Δ(*ara-leu*)7697 *galU galK rpsL (StrR) endA1 nupG*	Invitrogen
*Salmonella enterica *serovar Typhimurium		
SL1344	*xyl hisG rpsL*; virulent; Sm^R^	[30]
CMPG5602	SL1344 *ΔluxS *- deletion of the entire *luxS *CDS	[10]
CMPG5702	SL1344 *luxS*::*Km^R^*	[14]
CMPG5630	SL1344 *ΔluxS2 *- deletion of the 3' end of *luxS *CDS	This study
CMPG5628	SL1344 *Δhfq*	This study
JVS-01028	SL1344 *ΔrpoE::Cm^R^*	[34]
CMPG5643	SL1344 *ΔompA::Km^R^*	Phage lysate of J. Vogel
CMPG5648	SL1344 *ΔlamB*	This study

**Plasmids**		

pJV300	pZE12-luc based plasmid; P_LlacO_-rrnB terminator; short nonsense transcript; control plasmid; Amp^R^	[35]
pJV871.14	pZE12-luc based plasmid; LT2 MicA overexpression construct; MicA transcription driven from constitutive P_LlacO_, starting precisely at G_+1_; Amp^R^	[36]
pJV853.1	pZE12-luc based plasmid; LT2 anti-MicA expression construct; anti-MicA transcription driven from constitutive P_LlacO_; Amp^R^	J. Vogel, unpublished data
pKD3	Template for mutant construction; carries chloramphenicol-resistance cassette; oriRγ origin; Amp^R^	[31]
pKD46	P_araB_-γ-β-exo; temperature-sensitive lambda-red recombinase expression plasmid; oriR101 origin; Amp^R^	[31]
pCP20	Temperature-sensitive FLP recombinase expression plasmid; oriR101 origin; Amp^R^, Cm^R^	[31]
pFAJ1708	Derivative of RK-2; Amp^R^; Tc^R^; contains *nptII *promoter of pUC18-2	[32]
pCMPG5685	pFAJ1708 OmpA complementation construct	This study
pCMPG5687	pFAJ1708 LamB complementation construct	This study
pCMPG5638	pCS26-*Pac *plasmid carrying a transcriptional reporter fusion between the promoter of the *lsrACDBFGE *operon and *luxCDABE*	[10]

**Table 2:** Primers used in this study

Primer	Sequence	**Purpose**^**a**^
PRO-483	TTTCAGAATCGAAAGGTTCAAAGTACAAATAAGCATATAAGGAAAAGAGAGTGTAGGCTGGAGCTGCTTC	FW CMPG5628
PRO-484	AGCGGGGGCGATTATCCGACGCCCCCGACATGGATAAACAGCGCGTGAACCATATGAATATCCTCCTT	RV CMPG5628
PRO-487	TTTGCTGGCTTTATGCGCGACCACCTCAACGGTAACGGCGTTGAGATTATGTGTAGGCTGGAGCTGCTTC	FW CMPG5630
PRO-229	CGGCCATAAACCGGGGTTAATTTAAATACTGGAACCGCTTACAAATAAGACATATGAATATCCTCCTTA	RV CMPG5630
PRO-0472	TGATGTTTCCGAGGGGCTTGCGCCCCTCGTTACGTCAGATGACCATCGTACATATGAATATCCTCCTTA	FW CMPG5648
PRO-0473	CCATTCGCAGTTTTAGAAGGTGGCAGCGTTTAAAGAAAAGCAATGATCTCGTGTAGGCTGGAGCTGCTTC	RV CMPG5648
PRO-0101	ATTCTAGACTTTACATCGCCAGGGGTGCTCAG	FW pCMPG5685
PRO-0102	ATCTGCAGCGCTGAAAGGCGTTGTCATCCAG	RV pCMPG5685
PRO-0474	ATTCTAGACCATTCGCAGTTTTAGAAGG	FW pCMPG5687
PRO-0475	ATCTGCAGTCATCAGACCTGATGTTTCC	RV pCMPG5687
PRO-2993	CTCACGGAGTGGCCAAAATT	FW RT-qPCR MicA
PRO-2994	GACGCGCATTTGTTATCATCAT	RV RT-qPCR MicA
PRO-1150	AAACGCGGGCAACTTCAG	FW RT-qPCR *rfaH*
PRO-1151	GTCAGGCAACTTACCGCTTGT	RV RT-qPCR *rfaH*

^a ^FW: Forward primer; RV: Reverse primer

When appropriate, antibiotics were applied at the following concentrations: 25 μg/ml choramphenicol and 100 μg/ml ampicillin. Strains were grown as a biofilm using the peg system as previously described [10]. For accurate comparison of data between peg plates, wildtype *S. *Typhimurium SL1344 was included in every plate as a control and data analysis was performed relative to the wildtype SL1344 values. In all figures, results are shown as a percentage of biofilm compared to wildtype SL1344 (100%). Error bars depict 1% confidence intervals of at least three biological replicates and each biological replicate is the average biofilm formation of eight technical replicates.

### AI-2 measurement

To measure AI-2 production of specific *S. *Typhimurium strains, the reporter plasmid pCMPG5638 was electroporated to the strains of interest. This plasmid contains a transcriptional fusion of the *lsrA *promoter region to the *luxCDABE *luminiscence reporter gene operon of *Photorhabdus luminescens *[10]. In *S. *Typhimurium, the expression of the *lsr *operon is regulated by AI-2 levels, and therefore luminescence of strains carrying the reporter plasmid is a measure for AI-2 production. Overnight cultures of strains of interest, were diluted 1:100 in fresh LB medium and grown for approximately 4 h, shaking at 37°C. Then, luminescence was measured together with the optical density at 600 nm. Wildtype SL1344 and CMPG5602 - *luxS *deletion mutant - were used as positive and negative control strains, respectively.

### RT-qPCR analysis

For RNA isolation, strains were grown as a biofilm in round petridishes. An overnight preculture in 5 ml Luria-Bertani broth (LB) medium, was diluted 1:100 in 20 ml 1:20 diluted TSB medium (Bacto™ Tryptic Soy Broth from BD Biosciences, 30 g/l) (resulting in approximately 10^7 ^cfu/ml) and poured carefully into a round petridish. These petridishes were incubated non-shaking at 16°C for 24 h. After the medium was removed, cells from the biofilm were scraped from the plate in a mixture of 1 ml 1:20 TSB and 200 μl ice-cold phenol:ethanol (5:95) and transferred to a microcentrifuge tube which was immediately frozen in liquid nitrogen and stored at -80°C. For strain CMPG5602, which is unable to form a mature biofilm, cells were incubated under the same conditions, but removed from the medium by centrifugation. Subsequent steps were identical for all strains. Total RNA was isolated from the cells using the SV Total RNA Isolation kit (Promega). This kit also allows extraction of small RNA molecules. RNA isolation was performed according to the manufacturer's instructions except for the DNase treatment, which was separately performed using the TURBO DNA-free Kit (Ambion) according to the manufacturer's instructions. DNA contamination of the RNA samples was checked by PCR. RT-qPCR analysis was essentially performed as previously described [33] with some minor modifications. 1.5 μg of RNA was reverse transcribed using the RevertAid H Minus First strand cDNA Synthesis Kit (Fermentas). After dilution of cDNA, 5 μl of cDNA (2 ng/μl), 0.9 μl of each specific primer (20 μM) and 3.2 μl of RT-qPCR grade water (Ambion) were mixed with 10 μl of Power SYBR Green PCR Master Mix. *rfaH *showed an invariant expression between the strains tested and was used as a reference gene [34]. Wildtype SL1344 samples were routinely used as reference sample.

## Authors' contributions

GK participated in the design of the study and drafted the manuscript. DDC carried out part of the experimental work. KM participated in the design of the study. JV and SCJDK conceived the study, participated in its design and coordination and helped to draft the manuscript. SCJDK also performed part of the experimental work. All authors read and approved the final manuscript.
